# Antipsychotic Dosage and Frequency of Manic Episodes as Predictors of Metabolic Syndrome in Bipolar Disorder: A One-Year Follow-Up

**DOI:** 10.1192/j.eurpsy.2025.314

**Published:** 2025-08-26

**Authors:** R. Tekdemir, M. T. Ergün, H. A. Güler

**Affiliations:** 1 Psychiatry; 2 Child and Adolescent Psychiatry, Selcuk University Faculty of Medicine, Konya, Türkiye

## Abstract

**Introduction:**

Metabolic syndrome (MetS) is notably prevalent among individuals with bipolar disorder (BD). Despite numerous studies indicating an increasing MetS prevalence in this group over time, comprehensive investigations of associated risk factors remain limited.

**Objectives:**

This study aims to assess the prevalence and 1-year changes in MetS among BD patients. It also seeks to identify baseline clinical features that could predict the development of MetS during follow-up.

**Methods:**

The study included euthymic BD type 1 patients consecutively admitted between July 2023 and July 2024. MetS was diagnosed uaccording to NCEP ATP-III criteria at baseline and after one year. Patients without MetS at baseline were analyzed to evaluate the association between initial clinical characteristics and MetS presence at follow-up through logistic regression.

**Results:**

A total of 98 patients completed the baseline and follow-up assessments. The prevalence of MetS significantly increased from 29.6% to 51.0% over the 1-year naturalistic follow-up. Initially, there were no significant differences between the groups with and without MetS regarding demographics, illness characteristics, treatment types, comorbidities, and chlorpromazine equivalent dose. By the end of the follow-up period, 29 new MetS cases were diagnosed after excluding those initially identified. This group exhibited higher numbers of total episodes, more manic episodes, and greater hospitalization rates (p = 0.04,-2.067; p = 0.03, -2.193; p = 0.03, -3.207), with no significant differences in other demographic or clinical variables.In the logistic regression analysis, which controlled for age, gender, number of depressive episodes, and the use of lithium and valproate, the equivalent chlorpromazine dose (p = 0.04, OR: 1.003) emerged as a significant predictor of metabolic syndrome, while the number of manic or hypomanic episodes demonstrated a trend towards significance (p = 0.05).

**Conclusions:**

In conclusion, this study shows that the prevalence of MetS in patients with BD type-1 in Turkey increased from 29.6% to 51.0% over one year. Increased numbers of manic episodes and higher chlorpromazine doses were linked to the development of MetS. This underscores the importance of monitoring metabolic health, especially in patients with frequent manic episodes or high antipsychotic doses.

**Disclosure of Interest:**

None Declared

